# Determinants of childhood blood pressure using structure equation model: the CASPIAN–V study

**DOI:** 10.1186/s12872-020-01488-z

**Published:** 2020-04-22

**Authors:** Pooneh Angoorani, Shayan Mostafaei, Toktam Kiani, Hanieh-Sadat Ejtahed, Mohammad Esmaeil Motlagh, Gita Shafiee, Armita Mahdavi Gorabi, Mostafa Qorbani, Ramin Heshmat, Roya Kelishadi

**Affiliations:** 1 Chronic Diseases Research Center, Endocrinology and Metabolism Population Sciences Institute, Tehran University of Medical Sciences, Tehran, Iran; 2 Medical Biology Research Center, Health Technology Institute, Kermanshah University of Medical Sciences, Kermanshah, Iran; 3 Rheumatology Research Center, Tehran University of Medical Sciences, Tehran, Iran; 4 Obesity and Eating Habits Research Center, Endocrinology and Metabolism Clinical Sciences Institute, Tehran University of Medical Sciences, Tehran, Iran; 5 Department of Pediatrics, Ahvaz Jundishapur University of Medical Sciences, Ahvaz, Iran; 6 Non-communicable Diseases Research Center, School of Medicine, Alborz University of Medical Sciences, Baghestan Blvd, Karaj, 31485/56 Iran; 7 Endocrinology and Metabolism Research Center, Endocrinology and Metabolism Clinical Sciences Institute, Tehran University of Medical Sciences, Tehran, Iran; 8 Department of Pediatrics, Child Growth and Development Research Center, Research Institute for Primordial Prevention of Non-communicable Disease, Isfahan University of Medical Sciences, Isfahan, Iran

**Keywords:** Blood pressure, Path analysis, Children, Adolescents

## Abstract

**Background:**

Childhood hypertension is a predictor of later diseases, increases the risk for cardiovascular morbidity and mortality in adulthood and results in major economic burdens.

The purpose of this study was to investigate the direct and indirect effect of anthropometric, socioeconomic and lifestyle factors on blood pressure (BP) in a large population-based sample of children and adolescents using a path analysis.

**Methods:**

This multi-centric nationwide study was performed on students aged 7–18 years. Anthropometric indices and blood pressure were measured by standard methods and demographic data, socioeconomic status, dietary habits and health related behaviors were obtained using validated questionnaires. Path analysis was applied to evaluate the relationships among the study variables and to implement the subsequent structural modeling.

**Results:**

Totally, 7235 students (50.6% boys; the mean age 12.3 ± 3.1 years) were assessed. Systolic and diastolic BP positively correlated with age (r = 0.35 and 0.26; respectively), BMI (r = 0.06 and 0.04; respectively) and WC (r = 0.05 and 0.03; respectively). According to path analysis, age had significant direct effect on BMI, WC, and BP (β = 0.035, 0.043 and 0.345; respectively), which was greater for BP. BMI and WC had the greatest direct effect on BP (β = 0.05 and 0.03; respectively). Education level, subjective health complaints, health-related behaviors and dietary habits had positive direct effects on BP (β = 0.036, 0.030, 0.018 and 0.017; respectively). Socioeconomic status and positive changes in diet had negative indirect effect on BP (β = − 0.001 for both).

**Conclusion:**

Our findings strengthen the importance of weight and body composition in BP control. It is suggested to improve diet and health related behaviors especially in families with low socioeconomic position.

## Background

High blood pressure that originates early in life is a predictor of later diseases, increases the risk for cardiovascular morbidity and mortality in adulthood and results in major economic burdens [1, 2]. The estimated prevalence of hypertension in children is around 3.5%, which increases progressively with age [3]. High blood pressure was reported about 4% in Canadian children and adolescents [4]. The prevalence of hypertension was reported 6.82% among Iranian school children by considering the additive prevalence of pre hypertension and hypertension; isolated high systolic and diastolic blood pressure was documented in 4.17 and 4.33%, respectively [5]. Both genetic and environmental factors play roles in pathology of hypertension in children and adolescents [6]. The high prevalence of hypertension in kids is considered as a main threat for the widespread occurrence of overweight and obesity in the past few decades. Therefore, paying more attention to the factors associated with childhood hypertension would be helpful for primary prevention of its complications in adulthood.

Although numerous reports have documented higher blood pressure parallel to the rise in childhood obesity [7], it requires to be demonstrated at a population level, with considering effective environmental variables including socio economic status (SES), dietary pattern, physical activity and other heath related behaviors. Multivariate multistage model like path analysis (structural equation modeling) is more appropriate method for analyzing the relationship between behavioral and biological factors with blood pressure. This model is more reasonable than multiple regression with its single dependent variable and has significant advantages for its extreme power in evaluating the interplaying direct and indirect effects between several predictor and outcome variables [8]. We conducted this study aimed to better understand the direct and indirect associations among anthropometric, socioeconomic and lifestyle factors with blood pressure in children and adolescents**,** using path analysis method. The results of this study would be helpful for primary prevention of hypertension and consequent complications later in adulthood.

## Methods

### Study population and sampling framework

This survey was performed in urban and rural regions of Iran in 2015 as the fifth national study of a school-based surveillance program entitled the Childhood and Adolescence Surveillance and Prevention of Adult Non-communicable disease (CASPIAN-V) study. The study was conducted on students in primary and secondary schools aged 7–18 years. Selection of 14,286 students was done by multistage, stratified cluster sampling method from 30 provinces of the country (48 clusters of 10 students in each province). Descriptions of sampling and operation details of the study has been published previously [9]. The Research and Ethics Council of Isfahan University of Medical Sciences (code: 194049) ratified the study protocol. Written and verbal agreements were taken from all the parents and students, respectively.

### Questionnaires

Two sets of the questionnaires were completed for students and their parents. The validate and reliable questionnaires were obtained from Global School Student Health Survey (GSHS) and translated to Persian [10]. The validity and reliability of the questionnaires has been assessed previously [11].

The student’s questionnaire, filled out by trained staff, had questions regarding the body image, and psychosocial environment of school, dietary habits, life-style habits and violence behavior. A team of health care professionals monitored all process.

The parent’s questionnaire, completed by trained interviewers, included questions about family composition, economic and socio-demographic factors and genetic determinants (family history of hypertension, diabetes, obesity), past history of student (birth weight, breastfeeding, type of complementary food), family dietary habits were included in the parent’s questionnaire.

### Anthropometric measurements

Examinations were performed under standard protocols using calibrated instruments. The project team measured height, weight, and waist circumference using standard protocols which were described previously in details [12]. Body mass index (BMI) was calculated by dividing weight (kg) to height squared (m^2^). We used the WHO growth charts to categorize BMI [13]. Blood pressure was measured in the sitting position on the right arm using a standard mercury sphygmomanometer. It was measured two times with 5-min interval and the average was registered [14].

### Factor definition

The method and items used for calculating family socioeconomic status (SES) were approved previously in the International Reading Literacy Study (PIRLS) [15]. Using principal component analysis, some variables/items including family assets (including house, car and computer), parental education and occupation, as well as the school type (private/public) were summarized in one main component for constructing family SES. Students were classified in low, moderate and high SES based on the score of this component.

For the health-related behaviors (HRB) factor, a validated questionnaire was used for estimating physical activity (hours/day). It was classified as the mild, moderate, vigorous, and extremely vigorous activities. The screen time (ST) behaviors were defined as the sum of the number of hours per day that participants spent watching television (TV), working with personal computer, or playing electronic games [16].

Subjective health complaints (SHC) were assessed by asking children to report the frequency of their experiences of a variety of emotional (feeling low, irritability, feeling nervous, difficulty in getting to sleep) and physical symptoms (headache, stomach ache, backache, feeling dizzy) during the past 6 months. Response options were ‘about every day’, ‘more than once a week’, ‘about every week’, ‘about every month’ and ‘rarely or never’. Then responses were categorized at weekly or more versus rarely or never. Items within the scale have shown an adequate content validity and test–retest reliability [17].

Dietary habits (DH) were assessed by asking children to report the eating pattern of items included dairy products, animal protein, plant protein, fast foods, salt, fatty snacks, vitamins/mineral supplement, sweets/candies, vegetables, fruits and carbohydrates [18]. History of hypertension, hyperlipidemia, diabetes, obesity, osteoporosis, heart attack or stroke and cancer in family members was considered as the positive family history (PFH) factor and reduction in consumption of high-fat foods, liquid oil, fast foods, sugar and salt, and alcohol drinks was considered as the positive changes in diet (PCD). All of the mentioned factors considered as continuous variables.

### Statistical analysis

All continuous variables were checked for normality. Continuous variables were expressed as means (SD) or median (IQR) according to normal and non-normal distributions, respectively. Categorical variables were presented as number (percentage). Spearman correlation was used for evaluating the correlation between different demographic, anthropometric and physiological variables. Path analysis was applied to evaluate the direct, indirect and total effect of the study variables and to implement the subsequent structural modeling. The method of fitting of path modeling was least square (OLS). The preferred value of the fitness indices of models in path analysis including normed fit index, comparative fit index, goodness of fit index, and adjusted goodness of fit index is above 0.9. Regarding the root mean square error of approximation criteria, score ≤ 0.05 indicates a good fit, and score up to 0.08 means acceptable [19]. Software including SPSS-version 16 and Lisrel-version 8.8 were used for data analysis with the application of path analysis.

## Results

In this national population-based study, among of 14,286 participants whose information was collected, 7235 were assessed (50.6% boys; the mean age: 12.3 ± 3.1 years). Difference between mean age of boys (12.4 ± 3.1) and girls (12.2 ± 3.2) was not statistically significant (*P* > 0.05). The mean of BMI, SBP and DBP was 26.5 ± 5.1, 99.1 ± 13.1 and 63.8 ± 10.4, respectively. The details of socioeconomic status, subjective health complaints, health-related behaviors and demographic characteristics of the participants are presented in Table 1.

**Table 1:** Demographic, anthropometric and physiological characteristics of the participants: CASPIAN-V study

Characteristics	Categories	Descriptive statistics
Age (Year)^b^	–	12.28 ± 3.158
Age^a^	7–10 (Year)	4843 (33.9%)
	11–14(Year)	5600 (39.2%)
	15–18(Year)	3843 (26.9%)
Gender^a^	Boy	7235 (50.7%)
	Girl	7041 (49.3%)
Educational level^a^	Primary	8400 (58.8%)
	Secondary	5886 (41.2%)
Body Mass Index^b^	–	26.49 ± 5.079
DBP (mm/Hg) ^b^	–	63.83 ± 10.435
SBP (mm/Hg)^b^	–	99.14 ± 13.10
SES (score)^c^	–	5.66 ± 1.710
Waist circumference (cm)^b^	–	87.61 ± 14.751
SHC (Score)^c^	–	32.14 ± 19.91
HRB (Score) ^c^	–	11.12 ± 3.19
Area of residency^a^	Urban	10,200 (71.4%)
	Rural	4086 (28.6%)
Family history of chronic diseases^a^	No	1757 (12.3%)
	Yes	12,529 (87.7%)

^a^ indicated as N (%); ^b^ indicated as Mean ± SD; ^c^ indicated as median (IQR)

**Fig. 1:**
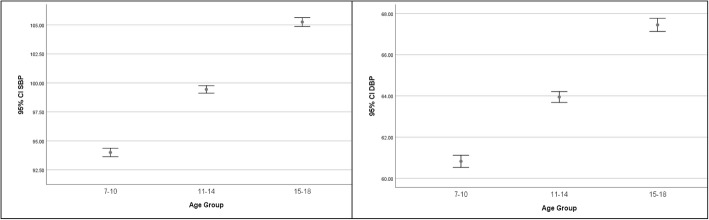
Mean (95% confidence interval) for systolic blood pressure (SBP) and diastolic blood pressure (DBP) against of age groups

*BMI* Body mass index, *DBP* Diastolic blood pressure, *SBP* Systolic blood pressure, *SES* Socioeconomic status, *WC* Waist circumference, *SHC* Subjective health complaints, *HRB* Health-related behaviors;

The mean SBP and DBP against age groups are shown in Fig. 1. The Spearman’s rank correlation matrix between different demographic, anthropometric and physiological variables are presented in Table 2. According to this table, SBP and DBP had slightly positive correlation with age (r = 0.35 and 0.26; respectively), BMI (r = 0.06 and 0.04; respectively) and WC (r = 0.05 and 0.03; respectively). However, no significant correlations observed between blood pressure and SES, SHC and HRB. WC showed a positive correlation with all studied variables (*P* < 0.05).

**Table 2:** Matrix of Spearman’s rank correlation between different demographic, anthropometric and physiological variables (*n* = 14,286): CASPIAN -V study

	BMI	DBP	SBP	SES	Waist	SHC	HRB
Age	0.04^*^	0.26^***^	0.35^***^	0.01	0.05^*^	0.02^*^	0.06^*^
BMI	1	0.04^*^	0.06^*^	0.17^**^	0.62^***^	0.03^*^	−0.001
DBP	–	1	0.66^***^	−0.013	0.03^*^	0.016	0.011
SBP	–	–	1	0.02^*^	0.05^*^	0.04	0.04^*^
SES	–	–	–	1	0.11^**^	0.05^*^	0.009
WC	–	–	–	–	1	0.06^*^	0.04^*^
SHC	–	–	–	–	–	1	−0.06^*^
HRB	–	–	–	–	–	–	1

**Fig. 2:**
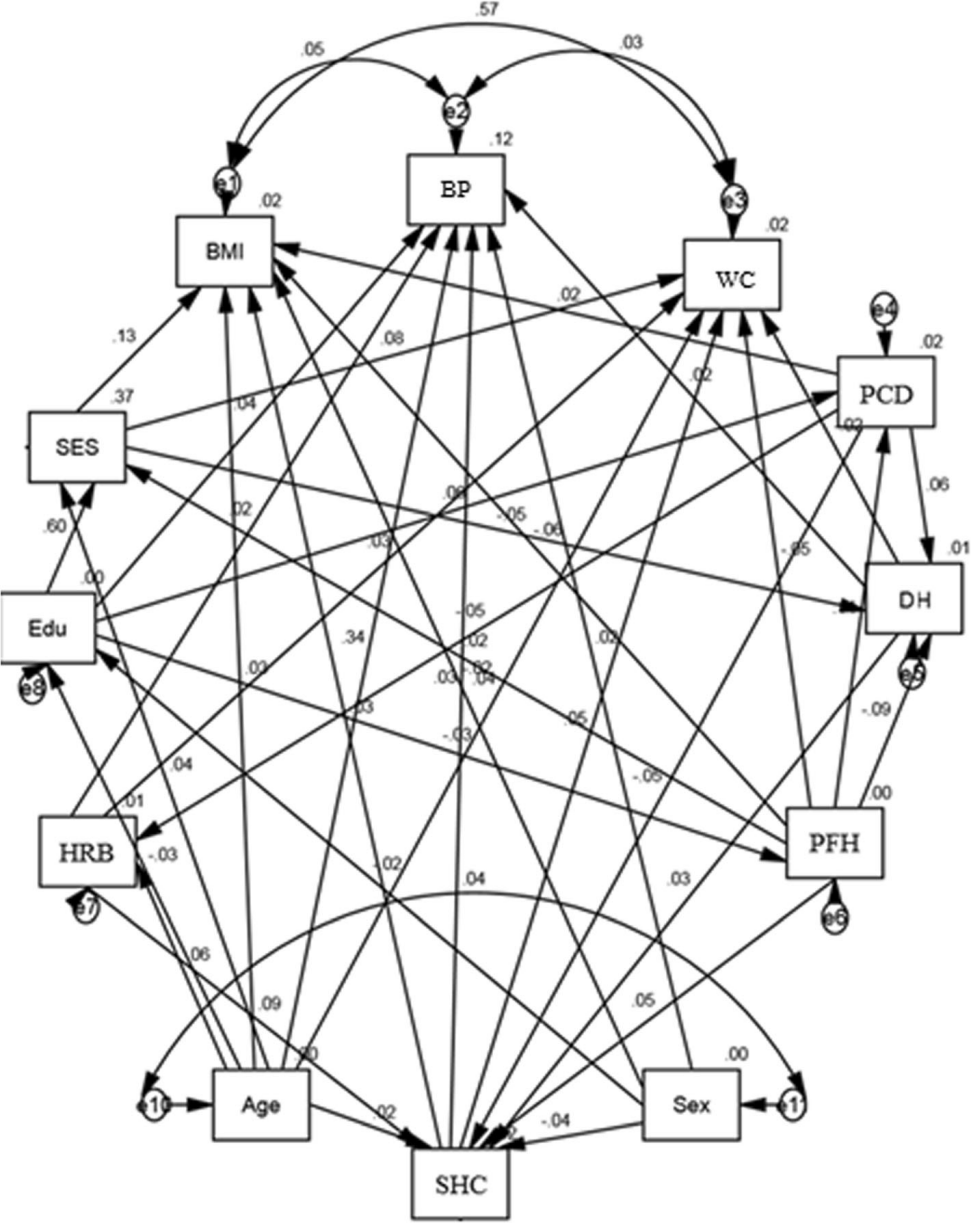
Path diagram for showing the associations between the study’s variables (BP: blood pressure, BMI: body mass index, WC: waist circumference, PCD: positive changes in diet, DH: Dietary habits, PFH: positive family history, SHC: subjective health complaints, HRB: health-related behaviors, SES: socioeconomic status

* *p* < 0.05; ** *p* < 0.01; *** *p* < 0.001; *BMI* Body mass index, *DBP* Diastolic blood pressure, *SBP* Systolic blood pressure, *SES* Socioeconomic status, *WC* Waist circumference, *SHC* Subjective health complaints, *HRB* Health-related behaviors;

According to the correlation matrix of the variables, path diagram is modeled as Fig. 2. The path standardized coefficients (β) are shown as direct, indirect and total effects of each variable on BMI, WC and BP in Table 3. Higher SES was directly associated with BMI and WC. The effect of SES on BMI and WC was greater than ones on BP. Age had significant direct effect on all three outcome variables (BMI, WC, and BP), which was greater for BP. Education level had positive direct effects on BP. Positive family history factor showed minor negative direct effects on BMI and WC (*P* < 0.05) and had no effect on BP. Dietary habits had slight positive direct effect on both BMI and BP which was greater for BP. Positive changes in diet had positive direct effect only on BMI. Education level, health-related behaviors and dietary habits in our path models had positive indirect effect on BMI, however, their direct effects on this variable did not reach the significant threshold. Education level, SHC, HRB and DH had positive direct effects on BP (β = 0.036, 0.030, 0.018 and 0.017; respectively). SES and PCD had small negative indirect effect on BP (β = − 0.001 for both).

**Table 3:** Direct, indirect and total effect obtained by path analysis

Variables	BMI			WC			BP		
	Direct	Indirect	Total	Direct	Indirect	Total	Direct	Indirect	Total
Sex	−0.022	−0.003	− 0.025	0.0	− 0.003	−0.003	0.020	−0.002	0.018
Age	0.035	0.003	0.038	0.043	0.005	0.048	0.345	0.001	0.346
SES	0.133	0.0	0.133	0.080	0.001	0.081	0.0	−0.001	−0.001
Edu	0.0	0.083	0.083	0.0	0.049	0.049	0.036	−0.001	0.035
SHC	0.027	0.0	0.027	0.045	0.0	0.045	0.030	0.0	0.030
HRB	0.0	0.002	0.002	0.031	0.004	0.035	0.018	0.003	0.021
DH	0.0	0.001	0.001	−0.025	0.001	−0.024	0.017	0.001	0.018
PFH	−0.057	0.002	−0.055	−0.056	0.005	−0.051	0.0	0.0	0.0
PCD	0.024	−0.001	0.023	0.0	−0.004	−0.004	0.0	−0.001	− 0.001

Data presented as path standardized coefficients (β) for each cell; All of the path standardized coefficients are statistically significant (*P*-values < 0.05); *BP* Blood pressure, *BMI* Body mass index, *WC* Waist circumference, *PCD* Positive changes in diet, *DH* Dietary habits, *PFH* Positive family history, *SHC* Subjective health complaints, *HRB* Health-related behaviors, *SES* Socioeconomic status

There are a variety of fit indices to evaluate the model. All of them indicated that the model was acceptable fitted. The results of model fitness with accepted range for evaluating the validity of the model are characterized in Table 4.

**Table 4:** The results of fitness of model indices

Goodness of fitness	Model	Accepted Range
Normal Theory Weighted Least Squares Chi-Square	χ^2^/*df*=1.96 *P*-value< 0.001	< 2
Akaike information criterion (AIC)	154.115	–
Root Mean Square Error of Approximation	0.008	< 0.08
Goodness of Fit Index (GFI)	0.999	> 0.95
Adjusted Goodness of Fit Index (AGFI)	0.998	> 0.90
Parsimony goodness of fit index (PGFI)	0.320	–
Root mean square residual (RMR)	0.107	> 0.08
Normed fit index (NFI)	0.997	> 0.90
Relative fit index (RFI)	0.992	> 0.95
Parsimony normed fit index (PNFI)	0.378	–

## Discussion

Our study clarified the obvious relations among anthropometric, socioeconomic and lifestyle factors with blood pressure in children and adolescents. To determine the true independent effects of anthropometric, socioeconomic and lifestyle factors on childhood blood pressure, we performed a path analysis that provides the full range of relations between variables. The path analysis revealed that among all variables (demographic and anthropometric) age had the greatest direct effect on BP, however, in the case of anthropometric variables, BMI and WC had the greatest direct effect. Moreover, by indirect-effects analysis, BP had negative correlations with SES and PCD while they had not direct effects on BP. The direct effects of weight and WC on BP were also reported in previous studies [20, 21]. High BP in childhood has increased with the epidemic of childhood obesity and reaching a prevalence of almost 5% of children and adolescents [22, 23]. Obesity has been known as a major risk factor of increased BP among children and adolescents. Furthermore, the latest studies reveal that in comparison to general obesity, central obesity has more powerful relationship with BP and other cardiovascular risk factors [24]. The BP depends on the blood flow and vascular resistance which in turn is determined by vasoconstriction and rigidity [25]. Studies in youths have found that blood flow pattern is influenced by weight and body composition. In the obese, high vascular resistance plays a major role as a cause of hypertension and hemodynamic load both on the heart and vessels, and also is considered as an important cardiovascular risk factor [21, 26, 27]. It was shown that that visceral fat is the primary etiological component underlying the effect of excess adiposity on development of hypertension, implicating the stronger association of BP with WC compared with BMI [28]. Adipose tissue is known to play important roles in vascular impedance and BP by releasing free fatty acids, thereby central adiposity seems to be especially deleterious because of the metabolic characteristics of fat stored in the intra-abdominal visceral compartment [29]. To find children at risk for increased BP could be based on recognizing the body composition characters. In this context, studies investigating the relationship of overall and abdominal obesity with elevated BP in children are necessary.

In current study, we also showed that the higher socioeconomic status and positive changes in diet can indirectly lead to beneficial effect on BP. The effect of SES indicators such as education attainment, household income or employment status on health is well established. A large body of research revealed that higher socioeconomic position individuals are healthier overall. Low education and low income are well-known causes of poor health and chronic medical diseases such as obesity, depression, and asthma [30–32]. The same rule seems to apply to BP. It was shown that higher socioeconomic position were protective against high BP [33].

The beneficial effect of healthier diet on BP, which was shown in this study, was also demonstrated in previous evidence. Dietary habit is considered one of the major modifiable determinants of hypertension and related chronic diseases in children and adolescents. Scientific evidences reveal the fact that a healthy diet pattern consisting of a daily intake of fruits and vegetables combined with a low consumption of salt, sugar, and saturated fat, in addition to industrially-produced-trans-fatty acids, is associated with a better cardio-metabolic profile both in adults and in children [34, 35]. High BP inchildren could be prevented or modified by correcting the inappropriate behaviors and unhealthy lifestyle [36]. Therefore, childhood is an important period for interventions to improve lifestyle to minimize long-term metabolic abnormalities.

Using path analysis for evaluating of the direct and indirect effect of anthropometric, socioeconomic and lifestyle factors on blood pressure in a large sample of Iranian children and adolescents is the strength of present study. While the cross-sectional design of our study represents a limitation for the emerged associations that cannot be considered causal. Moreover, the information on socioeconomic status and health-related behaviors was obtained by self-reporting which may affect the estimates by under- or over-reporting.

## Conclusion

Results of this study strengthen the importance of weight and waist circumference in blood pressure control. Our data also supports the need to improve dietary habits and health related behaviors especially in families with low socioeconomic position.

## Data Availability

The datasets used and/or analyzed during the current study are available from the corresponding author on reasonable request.

## Abbreviations

SES: Socio economic status
HRB: Health-related behaviors
SHC: Subjective health complaints
ST: Screen time
TV: Television
DH: Dietary habits
PFH: Positive family history
PCD: Positive changes in diet
BMI: Body mass index
WC: Waist circumference
BP: Blood pressure
SBP: Systolic blood pressure
DBP: Diastolic blood pressure
GSHS: Global school student health survey
